# Iatrogenic risks and maternal health: Issues and outcomes

**DOI:** 10.12669/pjms.301.4062

**Published:** 2014

**Authors:** Meharun-nissa Khaskheli, Shahla Baloch, Aneela Sheeba

**Affiliations:** 1 Dr. Meharun-nissa Khaskheli, MBBS, FCPS, Associate Professor, Department of Obstetrics & Gynaecology, Liaquat University of Medical & Health Sciences Jamshoro, Sindh, Pakistan.; 2 Dr. Shahla Baloch, MBBS, DGO, FCPS, Associate Professor, Department of Obstetrics & Gynaecology, Liaquat University of Medical & Health Sciences Jamshoro, Sindh, Pakistan.; 3 Dr. Aneela Sheeba, MBBS, DMRD, (FCPS), Assistant Professor, Department of Obstetrics & Gynaecology, Liaquat University of Medical & Health Sciences Jamshoro, Sindh, Pakistan.

**Keywords:** Iatrogenic risk factors, maternal morbidity, Management outcome, Mortality

## Abstract

***Objective:*** To observe acute maternal morbidity and mortality due to iatrogenic factors and outcomes.

***Methods: ***This observational cross sectional study was conducted at** i**ntensive care unit of Liaquat University of Medical and Health sciences Jamshoro from 1-January-2011 to 31-December-2012. In this study all the delivered or undelivered women who needed intensive care unit (ICU) admission due to management related life threatening complication referred from periphery or within this hospital were included, while those women who had pregnancy complicated by medical conditions were excluded. These women were registered on the predesigned proforma containing variables like Demographic characteristics, various iatrogenic risk factors, complications and management out comes. The data was collected and analyzed on SPSS version 20.

***Results: ***During these study period 51 women needed ICU care for different complications due to adverse effects of medical treatments. Majority of these women were between 20-40 years of age 41(80.39%), multiparous 29(56.86%), unbooked 38(74.50%), referred from periphery 39(76.47%), common iatrogenic factors were misuse of oxytocin 16(31.37%), fluid overload/cardiac failure 8(15.68%), blood reaction 7(13.72%), anesthesia related problems were delayed recovery 3(5.88%), cardiac arrest 2(3.92%), spinal shock 2(3.92%), surgical problems were bladder injury 5(9.8%), post operative internal haemorrhage 3(5.88%), 37(72.54%) women recovered and 14(27.45%) expired.

***Conclusion: ***The maternal morbidity and mortality rate with iatrogenic factors was high and majority of these factors were avoidable.

## INTRODUCTION

Iatrogenesis or an iatrogenic artifact “originates from a physician” is an adverse effect or complication resulting from medical treatment or advice including that of Psychiatrist, Therapist, Pharmacists, Nurses, Physician, and Dentist. Iatrogenesis is not restricted to conventional medicine, it can also results from complementary and alternative medicine treatments.

The term iatrogenesis means brought Forth by a healer (From the Greek Iatros, healer) as such in its earlier forms it could refer to good or bad effects. Iatrogenic conditions do not necessarily results from medical errors, such as mistakes made in surgery or the prescription or dispensing of the wrong therapy such as a drug. In fact intrinsic and some times adverse effects of a medical treatment are iatrogenic.

An integrated intervention based approach proved to be effective in finding severe acute maternal morbidity cases. Information on underlying causes and associated risk factors may improve prevention and treatment of obstetric morbidities, thus reducing feto maternal adverse effects and hospital expenditures.^1^

Leape LL and Brennan TA et al^2^^,^^3^ calculated the rate of error in the intensive care unit. First he found that each patient had an average of 178 “activities” (Staff/procedure/Medical interactions) a day of which 1.7 were errors, which means a 1% failure rate. Analyzing why there is so much medical error Leape acknowledged the lack of reporting.

 One year later in 1995, a report in JAMA said that, “over a million patients are injured in U.S hospitals each year and approximately 280,000 die annually as a result of these injuries, therefore the iatrogenic death rate dwarfs the annual automobile accident mortality rate of 45,000 and accounts for more deaths than all other accidents combined.^4^

 In some instances, though, the reported delay in receiving care or inadequate care may not have been real but perceived to be so by the family. Nevertheless, the availability and quality of emergency obstetric care is a matter of great concern in the country; two other national studies have shown similar results.^5^^.^^6^

Pregnant women are vulnerable to volume overload as their blood volume is already increased. Moreover sodium and water retention can occur secondary to oxytocin administration during delivery. Iatrogenic fluid overload is the commonest cause of oedema near term. In the past, when hypertonic saline was used to induce abortion, volume overload due to its escape into maternal circulation was another iatrogenic cause of pulmonary oedema. Women with pre-existing congenital or rheumatic cardiac disorders may not tolerate the hemodynamic burden of pregnancy and may end up with pulmonary oedema. Peripartum cardiomyopathy is an idiopathic dilated cardiomyopathy seen in pregnancy. It is more commonly seen in underdeveloped countries where the incidence may be 10% of all pregnancies. Patients present during the last trimester or in the early months after delivery with symptoms of biventricular congestive heart failure. The mortality rate can be as high as 30%–50%. In survivors it reoccurs with subsequent pregnancies. Pulmonary oedema may occur in patients with hypertrophic obstructive cardiomyopathy and in obese hypertensive patients.^7^

The aim of this study was to promote awareness among health professionals with regard to acute life threatening maternal morbidities due to medical error and their underlying risk factors. This may help in prevention and treatment of maternal morbidities and mortalities as well as hospital expenditures.

## METHODS

During this study period all the pregnant women in labour or immediately after delivery who had any life threatening medical or surgical management related complication resulted in intensive care admission were included after taking informed written consent, while the women with other directly related obstetrical problems like ante partum, post partum haemorrhage, pregnancy with various medical disorders were excluded.

The sample size was calculated by empirical way, confident interval 95%, prevalence 3.5%, formula N= (z)^ 2^ (pq)/e^2^ sampling procedure was simple random. Data was collected on predesigned proforma having variables like demographic characteristics, booking status, hospital or referred cases initial management given by the level of medical professional, iatrogenic medical or surgical management risk factors, intensive care management outcomes.

The data was analyzed on SPSS version 16.The result is presented in the form of simple percentage, Chi squire test was applied for qualitative type of data analyses P value 0.005 was considered as significant.

## RESULTS

Findings from this study showed that majority of the study population were between 21-40 years of age 41(80.39%), while 7(13.75%) women were above 41 years of age, high vulnerable group was grand multiparous women (Para 5 and above) 29(56.86%), booking status of these women was very poor as 38(74.50%) women were unbooked totally (p value 0.001), most of the affected cases 39(76.47%) of the study population got initial treatment at periphery and were referred here with complications, while 12 (23.52%) women were managed initially in this hospital and got complication, (P value 0.001). In referred complicated cases major population got initial treatment by the Para medical staff LHV/Nurses comprising 21(41.17%) women, medical officers managed 18 (35.29%) women in the hospital, initial management was given by the resident doctors on duty to 9 (17.64%) women, and senior residents managed 3 (5.88%) women (Table-I).

Frequent types of iatrogenic factor with medical management observed was misuse of oxytocin in 16 (31.37%) women, (P value 0.003), fluid over load/cardiac failure in 8 (15.68%) cases, (p value 0.003), blood reaction in 7(13.72%) cases, (p value 0.003), while with surgical management iatrogenic factors were bladder injury in 5(9.80%) cases, (p value 0.5), post operative internal haemorrhage and delayed blood reaction each in 3(5.88%) cases (P value 0.1), anesthesia complications were cardiac arrest in 2(3.92%) cases (p value 0.01), aspiration pneumonia in 1(1.96%) case (p value 0.001)(Table-II). Management outcome seen was full recovery in 37(72.54%) women, while 14 (27.45%) women expired (p value 0.001) despite the management due to various complications (Table-III).

**Table-I:** Demographic characteristics N=51

*S/N*	*Demographic characteristics*	*No of cases*	*Percentage*	*Chi square test*	*P Value*
1	*Age:* a. up to 20 years b. 21-40 years c. 41 years and above	3 41 7	5.88 80.39 13.75	79.4117 37.6862 53.6862	
2	*Parity:* a. Para 0-1 b. 2-4 c. 5 and above	6 16 29	11.76 31.37 56.86	59.6470 14.1568 1.9215	0.05 0.5
3	*Booking Status:* a. Booked b. unbooked	13 38	25.49 74.50	24.5101	0.001
4	*Referral status:* a. Referred from other places b. within hospital	39 12	76.47 23.52	28.5882	0.001
5	*Level of medical person dealing:* a. Paramedics LHV/Nurses b. medical officer c. resident doctor d. senior resident	21 18 9 3	41.17 35.29 17.64 5.88	3.1764 8.8235 42.7058 79.4111	0.5 0.5

**Table-II:** Iatrogenic factors N=51

*S/N*	*Iatrogenic factors*	*No of cases*	*Percentage*	*Chi square test*	*P Value*
1	*Medical 33 (64.70%)* a. Blood reaction b. drug reaction c. fluid overload/cardiac failure d. misuse of oxytocin	7 2 8 16	13.72 3.92 15.68 31.37	21.8787 50.9696 17.5151 0.0606	0.003 0.003 0.003
2	*Surgical 18 (35.29%)* A. Anesthesia a. Aspiration pneumonia b. cardiac arrest c. delayed recovery d. spinal shock B. Surgical trauma a. Bladder injury b. intestinal perforation c. post operative internal haemorrhage	1 2 3 2 5 2 3	1.96 3.92 5.88 3.92 9.80 3.92 5.88	28.4444 21.7777 16 21.7777 7.1111 21.7777 16	0.001 0.01 0.1 0.01 0.5 0.01 0.1

**Table-III:** Management outcome N=51

*S/N*	*Management outcome*	*No of cases*	*Percentage*	*Chi square test*	*P Value*
1	Recovered	37	72.54	20.74	0.001
2	Expired	14	27.45		

## DISCUSSION

In this study the complications resulted with blood reaction were 7(13.72%) ,and with adverse drug reaction were 2(3.92%), in comparison with Bhatti S and Penna L study^8^ incidence of anaphylaxis was 2.7/100,000 a serious rapid onset allergic reaction results in hypoxia due to bronchospasm, cardiovascular collapse and possible coagulopathy. In our study this high complication rate can be controlled by taking care during blood transfusion by proper crossmatching, grouping and vigilant monitoring during transfusion. The iatrogenic factors with medical management lead to fluid overload/cardiac failure were 8 (15.68%). The haemodynamic stresses of pregnancy and labour may precipitate unexpected cardiovascular collapse.^8^ Pulmonary oedema results from fluid imbalance.^9^ The amniotic fluid embolism is the second leading cause of maternal mortality^10^, predisposing factors are mismanaged labour, use of uterine stimulants, presence of me conium in the amniotic fluid, advanced maternal age, multiparty and intrauterine fetal death.^11^ The misuse of oxytocin resulted in complications in 16(31.37%) cases. Lovold A et al. study^12^ also reported that misuse of oxytocin in obstetrics practice may leads to serious effects on the health of mother. The intravenous fluids should be restricted according to the need and injudicious use of syntocin should be avoided.

In this study the rate of anesthetic complication observed were high similar as reported by other study as anesthetic complications are the seventh leading cause of pregnancy-related mortality in the United States, accounting for 1.6% of all pregnancy-related deaths.  Although rare, anesthesia-related maternal mortality is potentially preventable.^13^ guiding principles for the practice of analgesia for labour, anesthesia for caesarean section and the management of obstetric emergencies, where the anesthetist also has a central role, are suggested.^14^ Anaesthetist should be properly trained according to the guidelines for anesthesia in emergency obstetric cases.

Examples of diagnostic failure are considered as a cause of inadequate medical attention in gynecological and obstetric practice and in the treatment of iatrogenic (instrumental) complications during and after endotracheal intubation.^15^ in a pregnant woman, any acute medical, surgical or traumatic non-obstetrical disease can have obstetrical consequences. The most often described risks are early pregnancy loss, intra-uterine fetal death, placenta abruption, direct fetal hurts, preterm labor, prematurity and its complications. Obstetrical complications can induce maternal and neonatal life-threatening risks during the management of an acute non-obstetrical disease in a pregnant woman, once the mother condition was stabilized; the obstetrician has to estimate the fetal consequences and to adapt his or her therapeutic attitude. He or she sets up the fetal and placental surveillance adapted to the obstetrical risks and decides on the duration of this surveillance.^16^ A good quality care plays a vital role in women^’^s health**.**

In this study the frequency of bladder injury was 5(9.80%) comparing with other study overall incidence of bladder injury was 0.13%. Women with a bladder injury were more likely to have a prior caesarean delivery, presence of adhesions during the procedure was greater risk for bladder injury, moreover presence caesarean deliveries should be counseled about the potential for significant surgical complications in repeat caesarean deliveries when discussing the indications for a primary elective caesarean delivery.^17^ Careful labour monitoring, proper and optimum decision of intervention and by skilled person will decrease complication rate.

Unexpected rapid death after delivery due to HELLP syndrome (HS) may become the subject of a forensic expertise. These include unexpected, sudden and fulminant course of this syndrome, which may confuse physicians. On the other hand these characteristics cast doubt on violent injury, diagnostic oversights or iatrogenic injuries. A definitive postmortem diagnosis of HS in questionable cases of maternal death and consecutive forensic expertise of suspected medical malpractice should be based on accepted laboratory criteria and characteristic histopathological alterations.^18^ In this study mortality rate was 14(27.45%) major cause of death were severe blood reaction, undiagnosed cardiac problem and problems due to anesthesia, as reported by other studies ^19^^.^^20^, poor quality of obstetric care services results in 8% of all maternal deaths attributed to iatrogenic causes.^21^

The Millennium Development Goals (MDGs) set very high targets for Women’s reproductive health. By fulfilling these targets by 2015, there will be decrease in maternal and infant mortality by improving collaboration between government and mission hospitals especially in less developed areas. We encourage healthcare professionals all over the world to advocate for women's reproductive rights and in this way help to achieve the MDGs by 2015.^22^ There is strong need of collaboration between government and private hospitals, maternity homes in under developed areas for availability and quality of obstetrics services to meet MGD targets.^23^

Improving surgical and anesthetic mortality in the developing world is a global health priority. Quality improvement processes have a role to play in addressing this need and are applicable to low-resource settings despite difficulties in their implementation. International initiatives to reduce perioperative mortality can be focused to support interventions at the level of individual departments, but this requires integration with existing local systems and an understanding of the specific needs of the institutions concerned. There is a small but growing evidence base for quality improvement in low resource settings, but this needs to be locally accessible to allow self-sustaining evidence-based quality improvement.^24^

## CONCLUSION

The major iatrogenic factors were errors in obstetrics care, negligiences in blood transfusions and anesthesia leading to severe consequences. Proper care and management plays an important role in the life and health of women.
